# Altered Transcription Factor Expression Responses to Exercise in Insulin Resistance

**DOI:** 10.3389/fphys.2021.649461

**Published:** 2021-04-07

**Authors:** Rocio Zapata-Bustos, Jean Finlayson, Paul R. Langlais, Dawn K. Coletta, Moulun Luo, Danielle Grandjean, Elena A. De Filippis, Lawrence Mandarino

**Affiliations:** ^1^Division of Endocrinology, Department of Medicine, University of Arizona, Tucson, AZ, United States; ^2^Center for Disparities in Diabetes, Obesity and Metabolism, University of Arizona Health Sciences, Tucson, AZ, United States; ^3^Department of Medicine, Mayo Clinic, Scottsdale, AZ, United States

**Keywords:** exercise, gene expression, transcription factors, insulin resistance, skeletal muscle

## Abstract

**Purpose:**

Insulin resistant muscle is resistant to gene expression changes induced by acute exercise. This study was undertaken to identify transcription factors that differentially respond to exercise in insulin resistance. Candidate transcription factors were identified from analysis of 5′-untranslated regions (5′-UTRs) of exercise responsive genes and from analysis of the 5′-UTRs of genes coding for proteins that differ in abundance in insulin resistance.

**Research Design and Methods:**

Twenty participants took part in this study. Insulin sensitivity was assessed by an euglycemic clamp. Participants were matched for aerobic capacity and performed a single 48 min bout of exercise with sets at 70 and 90% of maximum heart rate. Muscle biopsies were obtained at resting conditions, 30 min and 24 h after exercise. Global proteomics analysis identified differentially abundant proteins in muscle. The 5′-UTRs of genes coding for significant proteins were subjected to transcription factor enrichment analysis to identify candidate transcription factors. Q-rt-PCR to determine expression of candidate transcription factors was performed on RNA from resting and post-exercise muscle biopsies; immunoblots quantified protein abundance.

**Results:**

Proteins involved in mitochondrial function, protein targeting and translation, and metabolism were among those significantly different between lean and obese groups. Transcription factor enrichment analysis of genes coding for these proteins revealed new candidate transcription factors to be evaluated along the previously identified factors. Q-rt-PCR analysis of RNA and immunoblot analysis from pre- and post-exercise muscle biopsies revealed several transcription and growth factors that had altered responses to exercise in insulin resistant participants. A significant increase (EGR3 and CTGF) and decrease (RELA and ATF2) in the mRNA expression of transcription and growth factors was found after exercise in the lean group, but not in the obese participants.

**Conclusions:**

These results confirm findings of an association between insulin sensitivity and transcription factor mRNA response to exercise and show that obesity also may be a sufficient prerequisite for exercise resistance. Analysis of the muscle proteome together with determination of effects of exercise on expression of transcription factors suggests that abnormal responses of transcription factors to exercise may be responsible for differences in protein abundances in insulin resistant muscle.

## Introduction

The results of many studies reveal a variety of differences between skeletal muscle from insulin sensitive and insulin resistant individuals. Nevertheless, the significance to the pathogenesis of insulin resistance of these differences is unclear. One particularly intriguing difference that has received attention is the observation that transcriptional responses to acute exercise are related to insulin sensitivity but are independent of obesity. This concept derives from studies in which participants matched for body composition but differing in insulin sensitivity were found to have different gene expression responses to a single exercise bout, suggesting that insulin resistant muscle also is “exercise resistant” (De Filippis et al., 2008; McLean et al., 2015). We have defined exercise resistance as a reduced ability of a single bout of exercise to evince normal changes in gene expression in skeletal muscle from insulin resistant individuals. In this work, “exercise resistance” refers to this phenomenon relating to an acute, single bout of exercise. Understanding the mechanism underlying differences in the effects of exercise could have practical implications for the treatment of insulin resistance by physical activity. However, although our earlier studies showed a clear relationship between insulin resistance and lack of acute gene expression responses to insulin, it is unclear whether obesity itself also is associated with the impaired gene expression response to a single bout of exercise.

Regardless of its origin, it is unclear whether this phenomenon describes a globally lower molecular response to acute exercise or rather is specific to certain genes. Furthermore, the mechanism underlying the altered response to exercise is not clear. Recent transcriptomic studies revealed a correlation of lower insulin sensitivity with a lower mRNA response to exercise for many genes, including a number of transcription factors following an acute bout of exercise (McLean et al., 2015). McLean’s analysis of the 5′-untranslated regions (5′-UTR, −950 to +50 bp) of these differentially expressed genes provided candidate transcription factors that might underlie the overall differential responses in insulin resistance. That analysis of the differentially expressed genes showed an enrichment of binding motifs for transcription factors such as SP1, KLF4, NFKB, RELA, and EGR1.

Presumably, skeletal muscle mRNA responses to repeated bouts of exercise are followed by corresponding changes in protein abundance under normal conditions. Therefore, over time, an impaired response to repeated single bouts of exercise could result in different levels of protein abundance in resting skeletal muscle. There are many known protein abundance differences in skeletal muscle of healthy and insulin resistant participants, notably including many proteins involved in mitochondrial function and protein synthesis (Hwang et al., 2010; Lefort et al., 2010). One study examined protein abundance changes induced by 4 weeks of exercise training in muscle of overweight patients with type two diabetes mellitus and found a small number of proteins to be altered in response to exercise training (Hussey et al., 2013). These included proteins involved in energy metabolism, as expected. However, because changes in protein abundance occur on a longer time scale than changes in gene expression, it is difficult to determine directly if differential gene expression responses to exercise translate into protein abundance differences. Therefore, in this study we took an alternative approach to address this issue. Given that the acute mRNA response to exercise and chronic differences in the muscle proteome exist between lean, insulin-sensitive versus obese insulin-resistance participants, we hypothesized that transcription factor-DNA interactions are likely a significant contributor to these phenomena. We further hypothesized that these differences could be due to altered responses of the transcription factors that regulate responses to exercise in other genes. To identify such proteins, we performed a high-density global protein abundance analysis in muscle from lean, healthy participants and obese, insulin resistant individuals. We then analyzed the promoter regions of genes for these proteins to determine if there were common transcription factors that were over-represented in the genes for these proteins. Combining this new set of transcription factors with those candidates determined in our previous studies, we performed Q-rt-PCR on RNA from muscle from lean, insulin sensitive and obese, insulin resistant participants before and after acute exercise to identify the genes were differentially responsive to acute exercise.

## Materials and Methods

### Human Participants

**FIGURE 1:**
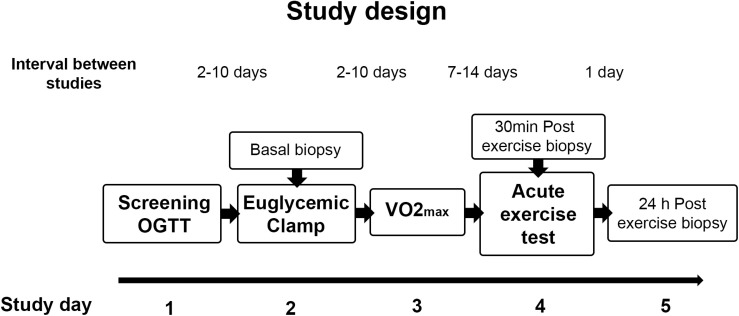
Study design. Participants were screened for inclusion criteria and an oral glucose tolerance test (OGTT) was performed. After completion of screening studies, participants performed a VO_2 *m*__*ax*_ test and a single bout of exercise, and insulin sensitivity was assessed by an euglycemic clamp. Muscle biopsies were taken at rest before the glucose clamp and 30 min and 24 h after completion of the single exercise bout.

Twenty sedentary volunteers took part in this study (eight lean control and twelve obese non-diabetic participants). The study was approved by the Institutional Review Boards of Arizona State University, the Mayo Clinic, and the University of Arizona. All studies were conducted at the Clinical Studies Infusion Unit (CSIU) at the Mayo Clinic in Arizona. Informed, written consent was obtained from all participants. None of the volunteers engaged in regular exercise (no more than three times per week for no more than 30 min) and none reported a change in body weight for at least 6 months before participation in the study. Participants were instructed not to exercise for 48 h before study and to maintain their usual diet. No participants were taking any medication known to affect glucose metabolism. A medical history, physical examination, 12-lead electrocardiogram, and a complete chemistry panel were obtained. Body composition was determined by bioelectrical impedance analysis (BioMarkers 2000). A 75-g oral glucose tolerance test was performed following the American Diabetes Association criteria (American Diabetes Association, 2020). After completing screening studies, the participants had measurements of insulin sensitivity (euglycemic clamp), a VO_2 *peak*_ test, and performed a single bout of exercise. Muscle biopsies were taken at rest before the glucose clamp and 30 min and 24 h after completion of an acute exercise bout. The study design is shown in Figure 1.

### Peak Aerobic Activity

Peak aerobic activity (VO_2 *peak*_) was determined using an electrically braked cycle ergometer and a Sensormedics model V29 Metabolic Measurement System (Sensormedics, Savi Park, CA, United States), as previously described (De Filippis et al., 2008). Briefly, exercise was started at a workload of 40 W and increased by 10 W/min until perceived exhaustion or a respiratory quotient of approximately 1.20 was reached. Heart rate and rhythm were monitored using a 12-lead electrocardiogram.

### Euglycemic, Hyperinsulinemic Clamp

Participants underwent a 2-h euglycemic, hyperinsulinemic clamp after a 10 h overnight fast, as previously described (DeFronzo et al., 1979). A stable isotope ([6,6-2H] glucose) was used to trace glucose metabolism, and steady state equations were used to calculate rates of glucose appearance and disposal during the last 30 min of a 2-h basal period and the final 30 min of a 2-h insulin infusion (80 mU∙m^–2^∙min^–1^). A percutaneous biopsy of the vastus lateralis muscle was obtained using a Bergstrom cannula an hour before the insulin infusion was started. This biopsy served as the basal specimen taken under resting, unstimulated conditions. Muscle biopsy specimens were immediately cleaned of blood and visible fat, frozen, and stored in liquid nitrogen until use.

### Acute Exercise Bout

All participants also underwent a single bout of exercise on a separate day after determination of VO_2 *peak*_, at least 1 week either before or after the euglycemic hyperinsulinemic clamp. Participants reported to the clinical research unit at about 7 AM after fasting overnight and were asked to exercise on a recumbent cycle (Schwinn 205P, Vancouver, WA, United States) for 48 min, not including warm-up, as previously described (see Supplementary Figure 1, Design of the acute exercise bout) (De Filippis et al., 2008; McLean et al., 2015). Participants warmed up by pedaling for about 5 min with no resistance, followed by four sets of exercise (8 min at 70% of max. heart rate, 2 min at 90% of max heart rate and 2 min of unloaded pedaling). After completing the exercise protocol, the participant was moved to a bed where a biopsy of the vastus lateralis muscle was performed within 30 min after completion of the exercise bout. The participants returned the next day after an overnight fast for a second post-exercise muscle biopsy.

### Muscle Processing for Proteomics Analysis

Muscle samples were homogenized, as previously described (De Filippis et al., 2008). For this analysis, 7 of the 8 lean and 11 of the 12 obese participants had sufficient biopsy material. Muscle samples were weighed while still frozen and were homogenized in ice-cold lysis buffer (1:10 wt/vol) containing 50 mmol/l Hepes (pH 7.6), 150 mmol/l NaCl, 20 mmol/l sodium pyrophosphate, 20 mmol/l β-glycerophosphate, 10 mmol/l NaF, 2 mmol/l EDTA (pH 8.0), 1% Igepal CA-630, 10% glycerol, 1 mmol/l MgCl_2_, 1 mmol/l CaCl_2_, 2 mmol/l sodium orthovanadate, 10 mg/l leupeptin, and 2 mmol/l phenylmethylsulphonyl fluoride. Homogenates were incubated on ice for 30 min and then centrifuged at 15,000 *g* for 20 min at 4°C. The supernatants were collected, and protein concentrations were measured by the Lowry method. Supernatants were stored at −80°C until use.

### Mass Spectrometry

Muscle lysate proteins were resolved by one-dimensional SDS-PAGE (Hwang et al., 2010). Each lane was cut into seven slices for the analysis. HPLC-ESIMS/MS was performed in positive ion mode on a Thermo Scientific Orbitrap Elite Velos Pro hybrid mass spectrometer fitted with an EASY-Spray Source (Thermo Scientific, San Jose, CA, United States). NanoLC was performed using a Thermo Scientific UltiMate 3000 RSLCnano System with an EASY RSLC C18 LC column (Thermo Scientific, 50 cm × 75 μm inner diameter, packed with PepMap RSLC C18 material, 2 μm, cat. # ES803). Spectra were acquired using Xcalibur, version 2.1.0 (Thermo Scientific). A “top 15” data-dependent MS/MS analysis was performed (acquisition of a full scan spectrum followed by collision-induced dissociation mass spectra of the 15 most abundant ions in the survey scan). Dynamic exclusion was enabled. Tandem mass spectra were extracted from Xcalibur “RAW” files and charge states were assigned using the ProteoWizard 2.1.x msConvert script using the default parameters. The fragment mass spectra were then searched against the human SwissProt_2015_12 database (20,194 entries) using Mascot (Matrix Science, London, United Kingdom; version 2.5.1). The search variables that were used were: 10 ppm mass tolerance for precursor ion masses and 0.5 Da for product ion masses; digestion with trypsin; a maximum of two missed tryptic cleavages; variable modifications of oxidation of methionine and phosphorylation of serine, threonine, and tyrosine. Cross correlation of Mascot search results with X! Tandem was accomplished with Scaffold (version Scaffold_4.4.0; Proteome Software, Portland, OR, United States). A Mascot ion score significance threshold of 25 was used. The mass spectrometry proteomics data have been deposited to the ProteomeXchange Consortium via the PRIDE (Deutsch et al., 2020) partner repository with the dataset identifier PXD023354 and 10.6019/PXD023354 (Zapata Bustos et al., 2021).

### mRNA Isolation and Analysis

**FIGURE 2:**
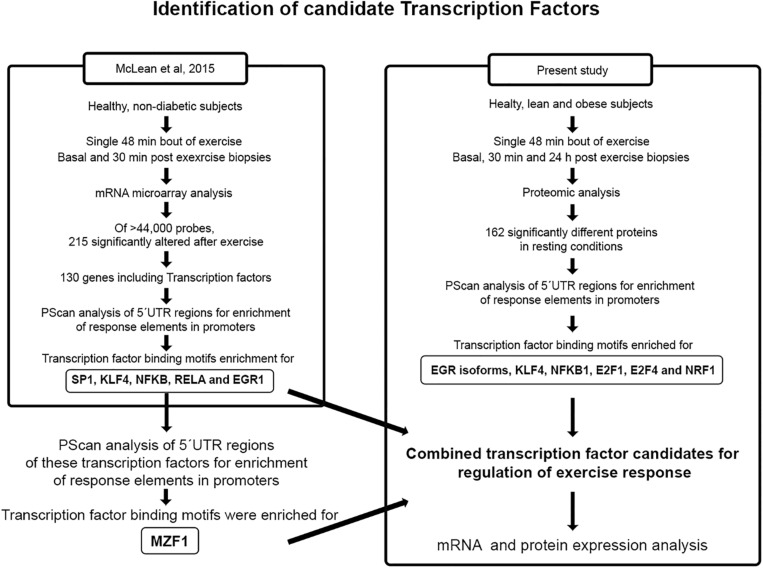
Identification of candidate transcription factors. The identification of transcription factors that are likely to regulate the molecular response to exercise was performed by PScan analysis of 5′UTR regions for enrichment of response elements in the promoters of genes alter by exercise (McLean et al., 2015) and proteins differentially expressed in resting conditions in lean and obese healthy participants.

RNA was isolated from portions of muscle biopsies as described (Richardson et al., 2005). Q-rt-PCR was performed as described (McLean et al., 2015), using primers listed in Supplementary Table 1. mRNA expression differences were determined by the 2^–Δ^ ^Δ^ ^*Ct*^ method (Livak and Schmittgen, 2001). Transcription factor enrichment analysis was conducted using PScan software (Zambelli et al., 2009) on the genes of proteins differentially expressed in the proteomic analysis at resting conditions, between lean and obese participants. In transcription factor enrichment analysis, the 5′-UTRs of genes are scanned for over-representation of putative consensus binding sites for transcription factors. A statistical analysis is done to compare enrichment in the set of genes of interest with random results from the genome. Significant enrichment for a given transcription factor means that these genes are more likely to be regulated as a set than a set of genes selected randomly from the genome. Whether individual genes in the set actually are regulated by such transcription factors would require experimental evidence. The resulting new candidates for transcription factors were also subjected to mRNA analysis. The analytical approach for the determination of transcription factors that are likely to regulate the molecular response to exercise is shown in Figure 2.

### Immunoblot Analysis

Immunoblot analysis was performed as described previously using biopsy specimens homogenized in the same manner as samples were prepared for proteomics analysis. Briefly, samples were loaded onto SDS-PAGE gels (4–20% gradient). Resolved proteins were transferred to PVDF membranes, blocked with 5% skim milk, incubated with primary (EGR3, Abcam ab232820, 1:500; MZF1, Thermo-Scientific PA5-41454, 1:500; NDUFA7, Invitrogen PA5-63693, 1:1000; NDUFA8, Abcam ab184952, 1:1000) and HRP-conjugated secondary antibodies, and finally developed with Western Lightning ECL Pro (PerkinElmer, Inc.).

### Data Analysis and Statistics

Differences in exercise-induced mRNA expression over time, between lean and obese participants were analyzed statistically using two-way repeated measures analysis of variance and *post hoc* tests differences were tested using Tukey’s multiple comparison test. Correlation analysis (Pearson’s correlation coefficients) was performed between insulin sensitivity and gene expression results. *In vivo* data (e.g., insulin sensitivity) values were compared using *t*-tests, with *P* < 0.05 being considered significant (GraphPad Prism 7.0, San Diego, CA, United States). Quantification of proteomic results were performed using the spectral abundance factor (NSAF) method as described previously (Hwang et al., 2010). Proteomic data values under basal conditions for lean and obese were derived by normalizing all values to the average value of the lean participants. When individual values are averaged, therefore, the mean value for the lean participants is 1.0, and the value for the obese participants reflects an average fold change relative to that. Comparison of values for protein abundance was performed using *t*-tests with Bonferroni correction. Gene ontology over-representation analyses were conducted using Panther v11.1 (Thomas et al., 2003; Mi et al., 2013). Heat maps for differences in abundance of proteins determined by mass spectrometry were generated using Heatmapper (Babicki et al., 2016). Cluster analysis for the heatmap was performed using an average linkage method with Euclidean distance measures.

## Results

### Participant Characteristics

Groups of lean and obese participants were well-matched for age (Table 1). The obese group was significantly heavier and had higher BMI and fat mass than lean control participants. Fasting serum insulin levels and LDL cholesterol were significantly higher in obese than in lean participants (Table 1). Other clinical parameters were comparable between the groups.

**TABLE 1:** Participant characteristics.

*N*	8	12
Male/Female	6/2	6/6
Age (years)	39.8 ± 11.82	39.7 ± 9.2
Weight (kg)	69.7 ± 9.59	97.4 ± 16.55**
BMI (kg/m^2^)	22.3 ± 2.43	33.5 ± 2.78**
Fat mass (kg)	12.6 ± 5.12	34.1 ± 8.5**
Lean mass (kg)	57.1 ± 9.31	63.1 ± 13.39
Hb A1c,%	5.4 ± 0.19	5.6 ± 1.02
Fasting plasma glucose (mg/dL)	86.1 ± 5.78	94.8 ± 12.27
Serum insulin (μIU/mL)	4.53 ± 1.77	14.3 ± 9.34*
HDL cholesterol (mg/dL)	65 ± 16.23	54 ± 14.5
LDL cholesterol (mg/dL)	90 ± 16.23	113 ± 26.7*
Total cholesterol (mg/dL)	171 ± 24.78	186 ± 31.6
Plasma triglycerides (mg/dL)	80 ± 29	112 ± 55
Systolic blood pressure (mmHg)	120 ± 10	127 ± 12
Diastolic blood pressure (mmHg)	73 ± 12	82 ± 8
Resting heart rate (beats/min)	62 ± 8	69 ± 8
Waist (cm)	76.3 ± 18.73	97.4 ± 31.8
Hip (cm)	91.4 ± 22.27	108.8 ± 33.2
Waist/Hip Ratio	0.84 ± 0.07	0.90 ± 0.07

Data are presented as Means ± SD. *P < 0.05, **P < 0.01 vs. Lean Controls (unpaired *t*-tests).

### Obese Participants Are Insulin Resistant as a Group

**FIGURE 3:**
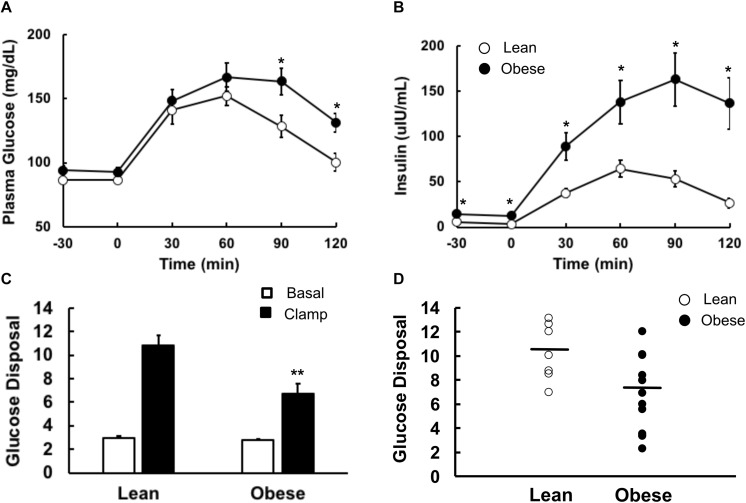
Glucose metabolism in lean and obese volunteers. All studies were done after an overnight fast, on separate days. **(A)** Plasma glucose and **(B)** plasma insulin concentrations during a 2-h 75 g oral glucose tolerance test. Lean participants are shown as open circles, obese as closed circles. **(C)** Basal (open bars) and insulin-stimulated (closed bars) rates of glucose disposal during an 80 mU⋅m^2^⋅min euglycemic, hyperinsulinemic clamp. **(D)** Basal (open bars) and insulin-suppressed (closed bars) endogenous glucose production. Data are shown as Means ± SEM. *P < 0.05, **P < 0.01 lean vs. obese.

Results of the oral glucose tolerance test showed significantly higher glucose levels (*P* < 0.05) in the obese group at 90 and 120 min compared to the lean controls, and plasma insulin concentrations were significantly higher in the obese group at all-time points (Figures 3A,B). A euglycemic, hyperinsulinemic clamp was used to determine insulin sensitivity. Plasma glucose concentrations during the final 30 min steady state portion of the glucose clamp were (93 ± 2 and 100 ± 3 mg/dL in lean and obese participants, respectively, *P* = NS), and plasma insulin concentrations during this period were (140 ± 11 and 186 ± 19 μU/mL, lean and obese, respectively, *P* = 0.05). Insulin-stimulated rates of glucose disposal during the final 30 min of the insulin infusion were significantly higher in the lean group compared to the obese (Figure 3C). Individual values for insulin-stimulated rates of glucose disposal, expressed in terms of mg/(kg fat-free mass) are shown in Figure 3D. Rates of endogenous glucose production and disposal were similar in lean and obese participants in the basal state and, after insulin stimulation, endogenous glucose production was suppressed completely in both groups, as expected using this insulin infusion rate (Figure 3D).

### Lean and Obese Participants Had Similar Exercise Capacity

The lean and obese groups were matched for maximum heart rate and VO_2 *peak*_ expressed relative to fat free mass (Table 2). Although VO_2 *peak*_ expressed relative to body weight was lower in the obese group, the difference did not achieve statistical significance. To elicit mRNA changes in skeletal muscle after exercise, the participants also underwent an acute, submaximal bout of exercise with muscle biopsies. Heart rate was monitored continuously, and the exercise intensity was adjusted as necessary to maintain the target heart rate. All participants reached their target heart rates during the exercise protocol, and there were no significant differences observed between groups (Table 2).

**TABLE 2:** Exercise characteristics of the participants.

	Lean	Obese
*N*	8	12
Maximum HR, beats/min	142 ± 17.59	155 ± 18
Maximum RER	1.24 ± 0.05	1.17 ± 0.11
*V*O_2 *peak*_ (ml⋅min^–1^⋅kg^–1^)	29.4 ± 10.5	22.2 ± 5.65
*V*O_2 *peak*_ (ml⋅min^–1^⋅kg FFM^–1^)	35.2 ± 10.9	35.0 ± 6.55
Maximum workload, W	164.3 ± 56	163.1 ± 41
Predicted 70% HR, beats/min	102 ± 11	108 ± 12
70% peak HR	99 ± 10	110 ± 17
Predicted 90% HR, beats/min	128 ± 16	139 ± 16
90% peak HR	128 ± 15	140 ± 18

*Values are means ± SD. Peak measurements are maximum values obtained during peak aerobic activity (VO_2 peak_) test. Maximum heart rate (HR) peak is the mean heart rate during the last minute of exercise during the VO_2 peak_ test. The maximum workload represents the highest workload reached and sustained, corresponding to the maximum HR and V_*O2 peak*_ observed during VO_2 *peak*_ test. There were no significant differences between the groups (unpaired *t*-tests) FFM, fat-free mass; RER, Respiratory Exchange Ratio.*

### Proteome Analysis of Resting Muscle

A total of 3466 proteins were assigned to at least one of the 18 participants (false discovery rate across all samples of 0.12%). The complete list of proteins assigned in the experiment is given in Supplementary Table 2 (Spectrum count and NSAF values for 3,466 proteins assigned in at least one participant with at least two exclusive spectra). Proteins present in at least half of the participants (9 of 18, total of 2,829 proteins) were analyzed further (see Supplementary Table 3, Spectrum count and NSAF values for 2,829 proteins assigned in at least half of the participants). Under resting conditions, 162 proteins were nominally statistically significantly different by unpaired, unadjusted *t*-tests in the two groups (see Supplementary Table 4, Protein fold difference values for 2,829 proteins assigned in at least half of the participants). Of these proteins, the majority were lower in muscle from obese, insulin resistant. These 162 proteins were submitted to Panther Gene Ontology analysis to determine which GO categories are over-represented in the data (Table 3). Classifications that were over-represented in the set of differentially abundant proteins included glycolysis, tricarboxylic acid cycle, fatty acid beta oxidation, oxidative phosphorylation, and others. The relative abundance of the 162 proteins for each individual is represented in Supplementary Figure 2 [Heat map of abundance of proteins that differed significantly (nominal significance, *P* < 0.05 by unpaired *t*-test), between lean and obese participants]. Cluster analysis confirms that for most protein abundance differences, a pattern of lower abundance in the obese participants prevails.

**TABLE 3:** GO classifications of proteins differing in abundance between lean and obese muscle.

Classification	Total	Observed	Expected	Fold enrichment	Bonferroni *P*
Glycolysis (GO:0006096)	34	11	0.64	17.13	2.26E-08
Tricarboxylic acid cycle (GO:0006099)	27	5	0.51	9.81	4.44E-02
Fatty acid beta-oxidation (GO:0006635)	34	6	0.64	9.35	1.32E-02
Oxidative phosphorylation (GO:0006119)	51	9	0.96	9.35	1.86E-04
Generation of precursor metabolites and energy (GO:0006091)	224	35	4.23	8.27	9.60E-19
Muscle contraction (GO:0006936)	154	18	2.91	6.19	4.06E-07
Respiratory electron transport chain (GO:0022904)	155	16	2.93	5.47	1.75E-05
Protein targeting (GO:0006605)	158	14	2.98	4.69	6.67E-04
Chromatin organization (GO:0006325)	263	22	4.97	4.43	2.72E-06
Fatty acid metabolic process (GO:0006631)	181	13	3.42	3.8	1.30E-02
Translation (GO:0006412)	323	18	6.1	2.95	1.41E-02
Cellular component morphogenesis (GO:0032989)	545	30	10.29	2.92	6.59E-05
Carbohydrate metabolic process (GO:0005975)	476	24	8.99	2.67	4.35E-03
Cellular component organization (GO:0016043)	1584	77	29.91	2.57	5.18E-12
Organelle organization (GO:0006996)	752	34	14.2	2.39	8.34E-04

*Proteins that differed significantly between lean and obese groups were subjected to Panther Pathway over-representation analysis. The expected number of proteins in a pathway is calculated assuming the same proportion of proteins in this pathway in the entire proteome would be present in a given subset of the proteome. Shown are classifications that were significantly and greater than two-fold enriched in the analysis.*

### Differentially Responsive Transcription Factors Are Significantly Enriched in Promoters of Genes Coding for Proteins That Differed in Abundance in Insulin Sensitive and Insulin Resistant Muscle

Next, we subjected the gene sequences for proteins that significantly differed in abundance between insulin sensitive and resistant muscle at baseline (resting conditions) to analysis of enrichment in predicted transcription factor binding sites. Analysis of the 5′-UTR’s of exercise responsive transcription factors (McLean et al., 2015) revealed binding sites for MZF-1 to be enriched, so this transcription factor was added to the list of potential candidates (Figure 2). The results of the Q-rt-PCR analyses of these genes are given in Table 4. Several of the transcription factors that had differential expression responses to exercise also were significantly enriched in the promoters of the genes coding for these proteins, including all three EGR isoforms, KLF4, and NFKB1. Other transcription factors also had enriched binding sites in the promoters of these genes, including E2F1, E2F4, and NRF1 (Figure 2).

**TABLE 4:** Acute effect of exercise on mRNA expression of transcription factors.

	Lean			Obese			Correlation with Rd
	Basal	30 min	24 h	Basal	30 min	24 h	r
EGR1	1.00	19.7 ± 10.3	6.8 ± 4.2	3.2 ± 3.4	3.8 ± 2.0	5.1 ± 5.6	0.35
EGR2	1.00	10.4 ± 8.3	10.7 ± 8.3	2.2 ± 1.5	2.4 ± 2.1	1.5 ± 1.2	0.16
EGR3**^†⁣†^	1.00	11.6 ± 4.3^†^	4.1 ± 1.7^†^	0.90 ± 0.31	1.3 ± 0.5	1.0 ± 0.7	0.31
RELA**	1.00	0.50 ± 0.12*	0.60 ± 0.14	1.1 ± 0.37	1.8 ± 0.41	1.5 ± 0.45	−0.40
NFKB1	1.00	0.57 ± 0.09	0.62 ± 0.06	8.4 ± 10.3	4.4 ± 4.4	1.1 ± 0.18	−0.01
CTGF**^†⁣†^	1.00	4.7 ± 1.0^†⁣†,^**	1.9 ± 0.50^†⁣†,^**	1.2 ± 0.23	1.0 ± 0.22	0.68 ± 0.21	0.36
MYC*	1.00	1.2 ± 0.29	1.2 ± 0.21	1.5 ± 0.53	2.7 ± 1.0	3.1 ± 1.5	−0.39
ATF3^†⁣†^	1.00	2.4 ± 1.1	0.76 ± 0.32	1.4 ± 0.70	2.3 ± 1.1	0.12 ± 0.06	0.05
SP1**^†⁣†^	1.00	0.57 ± 0.13^†⁣†,^**	0.72 ± 0.15	1.1 ± 0.18	1.5 ± 0.3	1.0 ± 0.17	−0.27
AP2	1.00	0.63 ± 0.10	1.2 ± 0.34	0.97 ± 0.25	1.2 ± 0.23	0.81 ± 0.17	−0.59^+^
KLF2^†^	1.00	1.7 ± 0.42	0.58 ± 0.12	0.87 ± 0.22	1.6 ± 0.24	1.6 ± 0.61	0.02
KLF4^†^	1.00	4.5 ± 1.6	1.0 ± 0.48	2.3 ± 1.3	3.9 ± 2.0	2.6 ± 1.5	0.11
KLF6vA*	1.00	1.0 ± 0.24	0.95 ± 0.23	1.1 ± 0.28	2.0 ± 0.48	1.6 ± 0.57	−0.28
KLF6vC	1.00	1.2 ± 0.35	1.3 ± 0.39	1.1 ± 0.31	2.0 ± 0.47	1.3 ± 0.50	−0.03
MZF1v1*	1.00	0.62 ± 0.14	0.90 ± 0.35	2.0 ± 1.1	2.5 ± 1.1	1.4 ± 0.48	−0.22
MZF1v3	1.00	1.2 ± 0.61	1.7 ± 0.92	1.4 ± 0.58	2.2 ± 1.2	0.97 ± 0.31	0.22
E2F1	1.00	2.5 ± 1.7	3.2 ± 1.9	1.4 ± 0.64	2.4 ± 2.0	0.80 ± 0.31	0.27
E2F3*	1.00	0.60 ± 0.12*	0.74 ± 0.11	0.85 ± 0.08	1.5 ± 0.36^†^	0.92 ± 0.17	−0.08
ATF2**^†⁣†^	1.00	0.39 ± 0.05^†⁣†,^**	0.46 ± 0.10^†⁣†,^**	0.82 ± 0.14	1.2 ± 0.13	1.2 ± 0.22	−0.53^+^

*, **P < 0.05, 0.01 vs obese, ^†,^^†⁣†^*P* < 0.05, 0.01 vs basal, *n* = 8 lean, *n* = 12 obese. Statistical analysis was performed using two-way repeated measures analysis of variance and *post hoc* tests differences were tested using Tukey’s multiple comparison test. Pearson’s correlations are given for the relationship between insulin-stimulated glucose disposal and the change in gene expression from basal to 30 min following exercise (^+^*P* < 0.05).

### Acute Exercise Differentially Alters Expression of Transcription Factors

**FIGURE 4:**
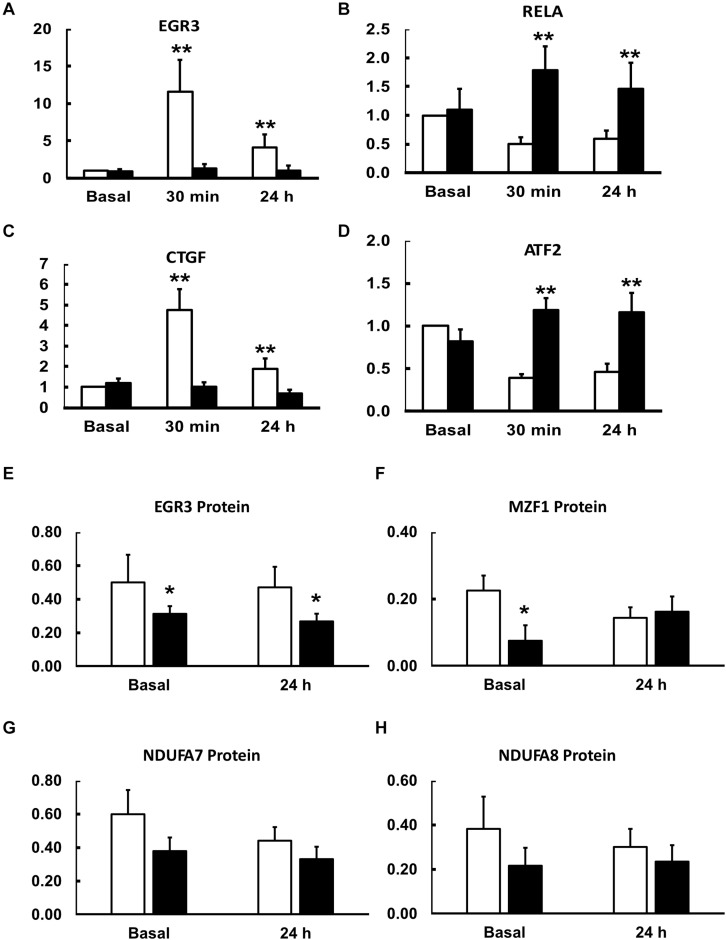
Effects of an acute exercise bout on mRNA for transcription and growth factors **(A–D)** and protein abundance basally and 24 h after exercise **(E–H)**. Muscle biopsies were taken at rest and 30 min and 24 h after a single bout of exercise (see section “Materials and Methods”). mRNA expression levels in lean (open bars) and obese (closed bars) participants were determined by Q-rt-PCR and all values were normalized to beta-actin expression and expressed relative to resting, basal values for lean, insulin sensitive participants. Data are shown as Means ± SEM for *n* = 8 and 12 for lean and obese participants, respectively. **P < 0.01 lean vs. obese.

Having identified candidate transcription factors first, from the results of our previous study, and second, from analysis of the 5′-UTRs of genes coding for proteins that differed in abundance in the proteomics analysis (Figure 2), we performed Q-rt-PCR on RNA isolated from resting muscle biopsies and those collected 30 min and 24 h after finishing the single exercise bouts. Several patterns of changes in transcription factor expression response to exercise emerged from this analysis (Table 4 and Figure 4).

**FIGURE 5:**
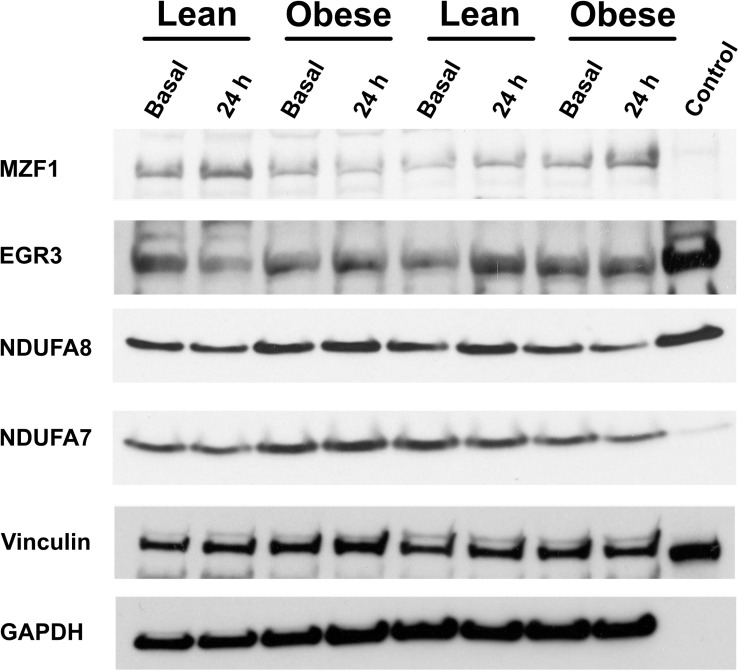
Representative immunoblots of proteins for which data is given in Figure 4.

First, some transcription factors, such as AP2, appeared to not be affected by exercise, nor did they differ in insulin resistance. Others, such as KLF4 and KLF2, were altered in expression by exercise, but did not differ in insulin resistant muscle. Another group, including EGR3 and CTGF (a growth factor rather than a transcription factor), responded briskly to exercise in the insulin sensitive participants but were unresponsive to exercise in insulin resistant muscle (Figures 4A,C). Others, like RELA and ATF2, had significantly lower expression after exercise in the insulin sensitive group but did not fall in the insulin resistant participants (Figures 4B,D). Interestingly, although the changes did not achieve significance, NFKB1 followed a similar pattern. Finally, some transcription factors responded more after exercise in the insulin resistant participants. These included MZF1 variant 1. Immunoblot analysis also was performed to determine the relationship between mRNA expression changes and changes in protein abundance. For these analyses, only basal, resting specimens and biopsies taken 24 h after exercise were used, the 30-minute post-exercise samples being deemed unlikely to show any protein changes because of the short time involved. These analyses were performed in subgroups of lean (*n* = 6) and obese participants (*n* = 8) who had sufficient remaining biopsy material. Two transcription factors, EGR3 and MZF1, had significantly lower protein abundance basally (Figure 4). EGR3 also had significantly lower abundance 24 h after exercise. To determine how changes in transcription factor abundance or gene expression might be translated into changes in mitochondrial proteins, further immunoblot analyses were performed. Figure 4 also shows these results for two protein subunits of Complex I of the electron transport chain, NDUFA7 and NDUFA8. Both were lower basally and after exercise in the insulin resistant patients. Representative immunoblots for these four proteins are shown in Figure 5.

To determine whether exercise responses across lean and obese participants were associated with insulin resistance, we performed correlation analysis between rates of insulin-stimulated glucose disposal determined during the glucose clamp (Rd) and the change from basal expression to mRNA expression determined 30 min after the exercise bout (the point of maximal change). For most genes assayed, the expression response to exercise was not related to insulin sensitivity (Table 4). This included genes such as EGR3, RELA, CTGF, and others which had significantly lower exercise responses in obese participants. One gene, AP2, had exercise responses that were significantly negatively associated with insulin sensitivity (*r* = −0.59, *P* < 0.05), but did not differ between the groups. The only gene that had significantly lower responses in obese participants as a group and had exercise responses that were negatively correlated with insulin sensitivity (*r* = −0.53, *P* < 0.05) was ATF2, whose mRNA significantly fell in response to exercise in lean but not obese participants.

## Discussion

The primary purpose of the present study was to determine whether some of the candidate transcription factors differed in their own response to exercise in insulin sensitive and insulin resistant muscle. This could provide additional evidence regarding the mechanism underlying insulin resistance. Many physiological, biochemical, and molecular differences between skeletal muscle from insulin sensitive and insulin resistant individuals have been described. One difference that has received recent attention is the lower gene transcription response to exercise in insulin resistance. Originally termed “exercise resistance,” this referred to an inability of insulin resistant muscle to briskly increase expression of the transcriptional coactivator PGC-1a and its downstream genes in response to an acute exercise bout (De Filippis et al., 2008; Dela and Stallknecht, 2010; Hernandez-Alvarez et al., 2010; Nilsson et al., 2010; Roberts et al., 2010; McLean et al., 2015; Stephens and Sparks, 2015). Recently, we showed that the muscle global transcriptome responds rapidly and widely to a single exercise bout in healthy participants (McLean et al., 2015). Moreover, for the genes that had significantly higher expression following acute exercise, analysis of their 5′-UTRs suggested enrichment in canonical binding sites for several transcription factors (McLean et al., 2015). We found that many of these transcription factors themselves had 5′UTRs that were enriched in binding sites for MZF-1, a transcription factor primarily known to be involved in regulating transcription in myeloid lineage precursors, and more recently in various cancers (Lenny et al., 1997; Nishiyama, 2006; Eguchi et al., 2015). Moreover, MZF-1 also has been implicated in myogenesis (Zhang et al., 2013) and transcriptional regulation of VEGF (Metzger et al., 2015). In addition to transcription factors that were identified previously as potential candidates for explaining altered gene expression changes in response to exercise in insulin resistance, we also reasoned that differences in protein abundance in insulin sensitive and insulin resistant muscle also might be explained by exercise resistance. Performing a global proteomics quantification analysis on insulin sensitive and insulin resistant muscle allowed us to again use transcription factor binding site enrichment analysis on genes coding for differentially abundant proteins. After all, previous studies were conducted mainly in participants with normal insulin sensitivity (McLean et al., 2015). Previous findings showed that insulin resistance itself was a sufficient prerequisite for the altered gene expression in response to exercise. However, in those studies, the participants had BMI’s on average in a range that was intermediate between obesity and leanness. In this study, the use of obese volunteers also allowed a determination of whether obesity, in addition to insulin resistance, also can be associated with the impaired response to exercise.

We performed Q-rt-PCR analysis to determine mRNA responses to a single exercise bout in lean, insulin sensitive and obese, insulin resistant non-diabetic volunteers. Of the total of 19 candidate transcription or growth factors that might be involved with the regulation of the exercise response, nine had significantly altered expression levels in response to exercise in the insulin resistant group. Broadly, the differentially expressed genes segregated into categories. Some transcription factors did not change in either group in response to exercise, including AP2, KLF6vC, MZF1v3, and E2F1. In others, exercise induced rapid rises in gene expression in insulin sensitive but not insulin resistant muscle. Genes in this category included transcription factors like EGR3, and the growth factor CTGF. EGR1 and EGR2 had similar patterns of expression changes after exercise, but the changes did not achieve statistical significance. For other genes, their expression fell after exercise in insulin sensitive but not insulin resistant muscle. These included RELA, SP1, E2F3, and ATF2. NFKB1 expression showed a similar pattern, but the differences were not significant. These finding show that exercise produces very specific effects on expression of transcription factors that together may coordinately regulate gene expression responses to exercise more broadly.

Some of the transcription factors that had altered response in insulin resistant muscle have connections to skeletal muscle function and development. EGR3 is essential for differentiation of muscle spindles, which are stretch proprioceptors that are involved in locomotor control and reflexes. A myocyte-specific EGR3 null mouse had major abnormalities in fusion of myocytes into muscle spindle myofibers (Oliveira Fernandes and Tourtellotte, 2015). A lower ability of exercise to induce increases in EGR3 expression could have implications regarding the function of insulin resistant muscle and its ability to sense stress. The growth factor CTGF already has been identified as a factor that may be involved in insulin sensitivity in human muscle (Richardson et al., 2005). CTGF is a mito-attractant that is involved in cell adhesion and fibrosis in many cell types, including myofibroblasts (Ramazani et al., 2018). CTGF expression rises after muscle contraction and exercise (Kivela et al., 2007; McLean et al., 2015) and is part of what appears to be a coordinated transcriptional response in muscle that involves a variety of extracellular matrix and associated genes that is activated by maneuvers that alter insulin sensitivity (Richardson et al., 2005; Bajaj et al., 2007; Forrester et al., 2014). The lack of change in CTGF expression following exercise in insulin resistant muscle could be a marker of exercise-induced changes that raise insulin sensitivity or could be part of the underlying mechanism. The differences in MYC expression in insulin resistant muscle could indicate a lower angiogenic response to exercise. Genes involved in angiogenesis, like VEGF, are highly responsive to exercise (McLean et al., 2015), and MYC regulate transcription of VEGFA (Dadiani et al., 2009; Song et al., 2013). Other genes with altered responses, like ATF3 and SP1 have broad effects on gene expression, and ATF3 is known to be increased in expression by exercise (McKenzie et al., 2011; Fernandez-Verdejo et al.,2017a,b; Beiter et al., 2018).

The group of transcription factors that fall in expression in insulin sensitive but not insulin resistant muscle after exercise may also be relevant to broader differences in insulin resistant muscle. The fall in RELA (p65 subunit of NFkB) and NFKB1 (p105 subunit) in insulin sensitive but not insulin resistant muscle could have major implications regarding myogenesis and muscle regeneration. Classical NFkB signaling inhibits myogenesis (Bakkar et al., 2008; Bakkar and Guttridge, 2010; Lu et al., 2012) so a fall in expression of proteins involved in this pathway could promote myogenesis. The present findings suggest that this process may occur less efficiently in insulin resistant muscle, and that thereby a single exercise bout may be less effective in producing beneficial effects. Finally, the expression of ATF2 also fell in response to exercise in lean, healthy but not obese participants, and the fall in ATF2 expression was negatively correlated with insulin sensitivity. ATF2 is required for the increase in PGC-1α that is induced by muscle contraction, via the p38 MAP kinase pathway (Akimoto et al., 2005; Kim et al., 2013). However, this involves phosphorylation of ATF2 protein rather than changes in mRNA expression (Akimoto et al., 2005). The significance of the corresponding contraction-induced fall in ATF2 mRNA expression is not clear but deserves further study as it may give insights into the differences between lean and obese or insulin sensitive and insulin resistant muscle.

Finally, MZF1 was identified by binding site enrichment analysis as a transcription factor that may regulate expression of other exercise-sensitive transcription factors. The present results show that expression of MZF1 is higher in insulin resistant muscle under resting conditions and significantly different in response to exercise than in insulin sensitive muscle. The significance of this difference is unclear since a role for MZF1 in skeletal muscle is unknown. MZF1 is an intriguing candidate for a gene that plays a major role in the exercise response in skeletal muscle, since it may regulate a number of exercise-responsive transcription factors and therefore would be a candidate for a master regulator of the acute gene expression response. The observation that MZF1 expression responds to exercise differ in insulin sensitive and resistant muscle, together with the enrichment of MZF1 binding sites in the promoters of differentially responsive transcription factors compels further study of the role of this factor in exercise. Additional studies will be needed to test this hypothesis.

The current results extend previous findings relating insulin resistance and exercise resistance to their relationship with obesity as well. We previously showed that insulin resistance is a sufficient prerequisite for lower exercise-induced gene expression responses in skeletal muscle. In this study, the results show that obesity *per se* also is sufficient to indicate the presence of exercise resistance. Because the majority of exercise-induced gene expression changes were not correlated with insulin sensitivity, it can be speculated that obesity, even in the absence of insulin resistance, is sufficient to cause an altered molecular response to exercise. In fact, the present findings suggest that the relationship between obesity and exercise resistance may be dominant.

There are limitations and caveats that apply to this study. First, because of the complexity of these studies with multiple visits, the sample size is somewhat limited. A second limitation is that of study design regarding timing and number of the biopsies. A biopsy taken 24 h after exercise probably was too long to see maintained changes in mRNA expression of genes, and this can be observed in Table 4. It might have been useful to perform a biopsy at some intermediate time, several hours after the biopsy taken 30 min after exercise. However, this itself presents design problems. If a biopsy were taken on the same day several hours after exercise, the participants would be entering the fasting, rather than postabsorptive state, and this could confound the results. By the same token, if the participants were fed a meal, this also could confound the results. It may be difficult to design the optimal study in humans regarding timing of muscle sampling. Third, it would have been of interest to determine nuclear localization information for transcription factors as well as using assays designed to measure actual transcription factor binding to promoter regions of genes. However, limited size of the muscle biopsies makes it difficult to perform all the assays in the same biopsies. Finally, it is possible that in this study, the obese participants were more sedentary than the non-obese patients. Arguing against this are that exercise characteristics such as VO_2 *peak*_, heart rate, and exercise workload are similar between the two groups. Even if there were more subtle differences, for example, if there were a lower anaerobic threshold in the obese participants, the design of the study is such that the acute exercise bout would have been conducted at a higher relative effort in the obese participants, which should have led to higher responses, not lower. It should be noted that none of the participants in the present study engaged in formal, regular exercise. Rather, “exercise” for these individuals derives from voluntary muscle contraction that occurs in their activities of daily living, such as walking, climbing stairs, housework, chores, or physical activities of a job.

The present findings add to existing evidence that differences in insulin sensitivity and body composition in skeletal muscle are intimately associated with differential molecular responses to exercise. The present findings also extend this to obesity, where it appears that obesity also may be a sufficient variable to predict exercise resistance. We speculate that regardless of whether these differences in exercise-mediated gene expression initially are causative or a result of insulin resistance or obesity, altered transcription and growth factor responses to exercise likely serve to maintain insulin resistance by reducing the efficiency by which an exercise bout maintains muscle function and regeneration.

## Data Availability Statement

The datasets presented in this study can be found in online repositories. The names of the repository/repositories and accession number(s) can be found in the article/Supplementary Material.

## Ethics Statement

The studies involving human participants were reviewed and approved by the Institutional Review Boards of Arizona State University, the Mayo Clinic, and the University of Arizona. The patients/participants provided their written informed consent to participate in this study.

## Author Contributions

RZ-B acquired, analyzed, and interpreted the data, and drafted and revised the manuscript. JF, DC, and ED acquired and analyzed the data, and revised the manuscript. PL acquired, analyzed, and interpreted the data, and revised the manuscript. ML analyzed and interpreted the data. DM acquired the data, and revised the manuscript. LM conceived and designed the work, analyzed and interpreted the data, and drafted and revised the manuscript. All authors approved the final version of the manuscript and agreed to be accountable for all aspects of the work in ensuring that questions related to the accuracy or integrity of any part of the work are appropriately investigated and resolved.

## Conflict of Interest

The authors declare that the research was conducted in the absence of any commercial or financial relationships that could be construed as a potential conflict of interest.
