# Supplementary material for: Altered Transcription Factor Expression Responses to Exercise in Insulin Resistance
Source: Front Physiol. 2021 Apr 7;12:649461. doi: 10.3389/fphys.2021.649461 (PMC8058368; doi:10.3389/fphys.2021.649461)
Supplement: Supplementary file 1 [file Table_1.DOCX]

| **Supplementary Table 1.** Primers used in Q-rt-PCR analyses | | |
| --- | --- | --- |
| **Gene** | **Forward primer** | **Reverse primer** |
| Beta-actin | AAACTGGAACGGTGAAGGTG | AGAGAAGTGGGGTGGCTTTT |
| EGR1 | CTAGTGAGCATGACCAACCC | AAACAGGTAGTCGGGGATCA |
| EGR2 | ACACCAAGATCCACCTGAGA | CCTTTGGGAGCTGGTGTATC |
| EGR3 | GGGGTTGTGAATTTCCAGGT | TACCAGAAGGAGCGAGGAAA |
| KLF2 | CCCAAACTGTGACTGGTATTTA | TATTTCTCACAAGGCATCACAA |
| KLF6 var A | CAAGGGAAATGGCGATGC | CTTTCGGAAGTGCCTGGTTA |
| KLF6 var C | GAAAATTGAGCTCCTCTGTCAC | GAAAAACACCTGTGTGCGT |
| ATF3 | GGTTAGGACTCTCCACTCAA | TAATAGACAGTAGCCAGCGT |
| MZF1 var1 | CCGACGGAGTTCTAA | AGTGTCTGCCCTTCTCTAAGG |
| MZF1 var3 | GACTACATCTGCCGGAAAGC | CCACCACGTTTTCCTCACTT |
| NFKB1 | CAATTGAAGTGATCCAGGCA | TTAGGGCTTTGGTTTACACG |
| RELA | GCCCCTATGTGGAGATCATT | AGCCATTGATCTTGATGGTG |
| SP1 | TTGAACATCCCCACTAGGTT | GGAACCACCCAAGAACATAC |
| CTGF | TCGATTAGACTGGACAGCTT | GGTGTTCAGAAATTGAGGCT |
| AP2 | CATGGCCAAACCTAACATGA | GCATTCCACCACCAGTTTAT |
| MYC | AACGACAGCAGCTCGCCCAA | AGGAGGTTTGCTGTGGCCTCCA |
| KLF4 | CCCCAAGATCAAGCAGGA | CATGAGCTCTTGGTAATGGAG |
| E2F1 | ACCGGGGAATGAAGGTGAAC | AGTGCTCTCACCGTCCTACA |
| E2F3 | GGGAAGTCGGGGACGTAAAA | TGGGTCAGGCACATAGCATT |
| ATF2 | AAAGTGCCGTTCAGCCTTTTC | GGCAAGAATACTTTCTAGCTGGT |
