# Supplementary material for: Altered Transcription Factor Expression Responses to Exercise in Insulin Resistance
Source: Front Physiol. 2021 Apr 7;12:649461. doi: 10.3389/fphys.2021.649461 (PMC8058368; doi:10.3389/fphys.2021.649461)
Supplement: Supplementary file 5 [file Image_1.PDF]

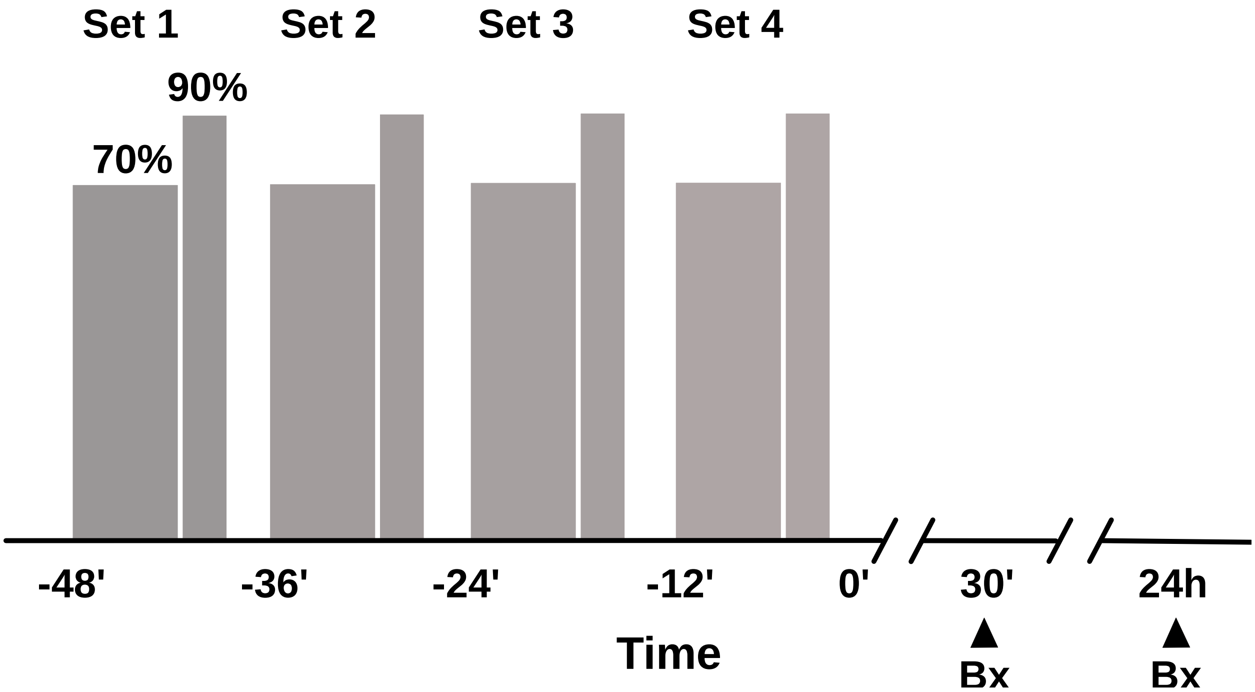

Figure S1. Design of the acute exercise bout. A baseline VO_{2peak} study was performed as described, and after warming up briefly, subjects exercise on a stationary recumbent cycle for 8 minutes at 70% of max heart rate, followed by 2 minutes at 90% of max heart rate and 2 minutes of unloaded pedaling. This set was repeated three more times, for a total of four sets. Muscle biopsies were performed 30 minutes after completing the last exercise bout and again 24 hours later (after an overnight fast). The resting biopsy was taken on a separate morning, on the day of the euglycemic clamp.
