# Supplementary material for: Altered Transcription Factor Expression Responses to Exercise in Insulin Resistance
Source: Front Physiol. 2021 Apr 7;12:649461. doi: 10.3389/fphys.2021.649461 (PMC8058368; doi:10.3389/fphys.2021.649461)
Supplement: Supplementary file 6 [file Image_2.PDF]

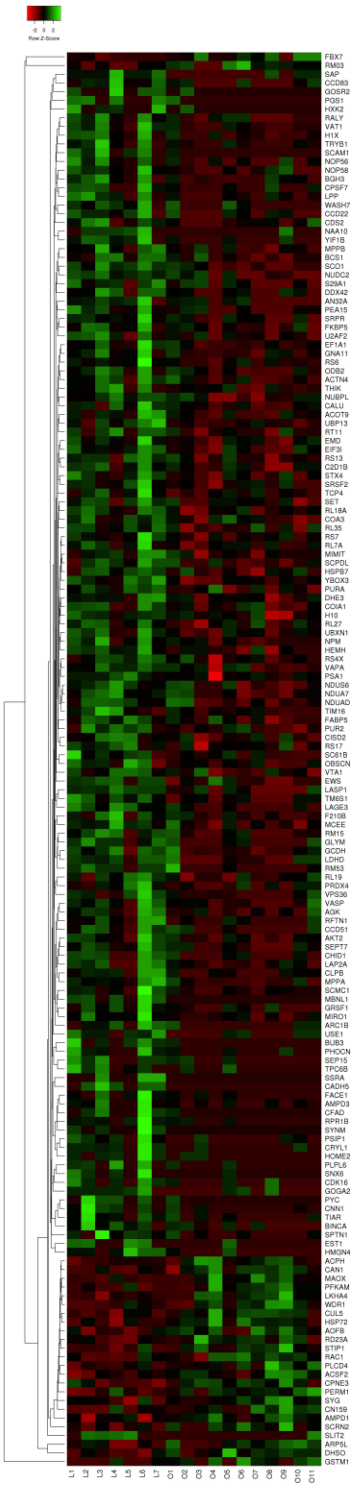

Figure S2. Heat map of abundance of proteins that differed significantly (nominal significance, P < 0.05 by unpaired t-test, between lean and obese subjects. Cluster analysis was performed using an average linkage method with Euclidean distance measures. Green indicates higher abundance, red indicates lower.
